# Furazolidone Increases Survival of Mice Exposed to Lethal Total Body Irradiation through the Antiapoptosis and Antiautophagy Mechanism

**DOI:** 10.1155/2021/6610726

**Published:** 2021-02-04

**Authors:** Shumei Ma, Zhao Jin, Yi Liu, Lin Liu, Hao Feng, Ping Li, Zhujun Tian, Minghua Ren, Xiaodong Liu

**Affiliations:** ^1^School of Public Health and Management, Wenzhou Medical University, Wenzhou, Zhejiang 325035, China; ^2^Department of Pharmacology, Shanghai Tenth People's Hospital, Tongji University School of Medicine, Shanghai 200092, China; ^3^Department of Urinary Surgery, The First Affiliated Hospital of Harbin Medical University, Harbin, Heilongjiang 150001, China; ^4^Zhejiang Provincial Key Laboratory of Watershed Science and Health, Wenzhou Medical University, Wenzhou, Zhejiang 325035, China

## Abstract

Exposure to total body irradiation (TBI) causes dose- and tissue-specific lethality. However, there are few effective and nontoxic radiation countermeasures for the radiation injury. In the current study, mice were pretreated with a traditional antimicrobial agent, FZD, before TBI; the protective effects of FZD on radiation injury were evaluated by using parameters such as the spleen index and thymus index, immunohistochemical staining of intestinal tissue, and frequency of micronuclei in polychromatophilic erythrocytes of bone marrow. The intestinal epithelial cell line IEC-6 was used to investigate the underlying mechanisms. Our results indicated that FZD administration significantly improved the survival of lethal dose-irradiated mice, decreased the number of micronuclei, upregulated the number of leukocytes and immune organ indices, and restored intestinal integrity in mice after TBI. TUNEL and western blot showed that FZD protected intestinal tissue by downregulating radiation-induced apoptosis and autophagy. Meanwhile, FZD protected IEC-6 cells from radiation-induced cell death by inhibiting apoptosis and autophagy. To sum up, FZD protected against radiation-induced cell death both in vitro and in vivo through antiapoptosis and antiautophagy mechanisms.

## 1. Introduction

With the rapid development of nuclear technology and the extensive application of nuclear medicine, ionizing radiation (IR) is increasing the health risk, and a series of pathological changes might occur and consequently cause tissue damage and even lead to death. Exposure to high-dose radiation induces acute radiation syndrome (ARS), manifested by DNA damage, cell death, organ and tissue damage, and so on [1]. Exposure to IR causes double-strand breaks (DBS) in DNA and consequently leads to genomic instability; simultaneously, the generation of reactive oxygen species (ROS) activates a myriad of molecular signaling pathways, leading to cell death and tissue damage [2, 3]. So far, it has been reported that IR triggers programmed cell death such as apoptosis, autophagic cell death, and mitotic catastrophe [4, 5]. High-dose radiation could induce ARS such as hematopoietic syndrome, bone marrow syndrome, gastrointestinal syndrome, and brain syndrome, in a dose-dependent manner.

Intestinal injury is a very common complication of radiotherapy. When the digestive system is severely damaged, acute gastrointestinal syndrome (AGS) is the predominant cause of death within the first week after IR exposure [6], and AGS is characterized by mucosal epithelium injury and the loss of gastrointestinal function. After irradiation, cell cycle arrest occurs in intestinal crypt epithelial cells and DNA synthesis is inhibited. Progressive necrosis and shedding of the epithelium of intestinal villi, atrophy of villi, and cell gap expansion could be detected.

Although research on a radioprotector is increasingly extensive, there are still problems to be solved. When AGS occurs, the routine treatment is aimed at reducing apoptosis of the gastrointestinal mucosal epithelial cell and restoring gastrointestinal function [6]. Chemical medicines are always accompanied by toxicities. It seems that Chinese herbal medicine and biological medicine are with fewer side effects, but the purification and identification of a simple compound are difficult. The optimal novel radioprotectors should be with higher efficacy, low toxicity, and ease of administration, which is important for acute IR injury. In this study, we choose a clinically used drug as a radioprotector candidate, to find out whether it could protect against radiation injury in vivo and in vitro.

Furazolidone, a traditional antimicrobial agent, has been extensively used to treat humans [7, 8]. FZD shows antibacterial effects on both Gram-positive and Gram-negative bacteria by interfering with bacterial oxidoreductase, causing metabolic disorders and death of the bacteria [9]. The current study also has shown the ability of FZD to inhibit proliferation and induce cell death and differentiation in human leukemia cells [10]. However, if FZD might be used as a radioprotector, it has not been fully elucidated. In the present study, the effect of FZD on TBI-induced tissue injury and lethality will be analyzed.

## 2. Materials and Methods

### 2.1. Reagents and Antibodies

Trypan blue solution (prod. no. T8154), phosphatase inhibitor cocktails 2 and 3 (prod. no. P5726 and P0044, respectively), and furazolidone (prod. no. V900742) were from Sigma-Aldrich (St. Louis, MO, USA). A protease inhibitor cocktail (ref. no. 11836 153 001) was from Roche Diagnostics (Basel, Switzerland). Primary antibodies anti-Atg5 (#2630), anti-beclin-1 (#3738), cleaved caspase-3 (#9661), Bcl-2 (#3498S), PARP (#46D11), H2AX (#2595S), LC3I/II (#4180S), BAX (#2772), and P53 (#9282) were purchased from Cell Signaling Technology (Danvers, MA USA), and anti-actin was purchased from Sigma-Aldrich (prod. no. A3853). Secondary antibodies goat anti-rabbit IgG- (H+L) HRP conjugate (cat. no. 170-6515) and goat anti-mouse IgG- (H+L) HRP conjugate (cat. no. 170-6516) were obtained from Bio-Rad Laboratories (Mississauga, Ontario, Canada).

### 2.2. Cell Culture

The intestinal epithelial cell line IEC-6 was obtained from the Beijing Cell Bank of the Chinese Academy of Sciences and periodically tested for the absence of mycoplasma. The cell line was cultured in Dulbecco's modified Eagle's medium mixture medium (Invitrogen Inc.) supplemented with 5% fetal bovine serum (Invitrogen Inc.), 0.01 mg/ml insulin (Absin), and 1% penicillin/streptomycin (cat. no. 10378016, Life Technologies) in a humidified 5% CO_2_,37°C incubator.

### 2.3. Animals

Male ICR mice with 20~24 g were purchased from the Center of Experimental Animals of Jilin University (Changchun, China). All mice were randomly divided into different groups. A normal diet and water could be provided for one week prior to the study allowed for acclimatization. The environment temperature was maintained at 25°C ± 2°C and a 12 h light/dark cycle. Animal treatments complied with a protocol approved by the Jilin University Institutional Animal Care and Use Committee.

### 2.4. Radiation

An X-ray generator (X-RAD 320 ix, Precision X-ray Inc., North Branford, CT, USA) was utilized to deliver radiation at a dose rate of 3 Gy/min. The distance between the source and the target was 70 cm.

### 2.5. TBI and FZD Treatment

Firstly, mice were exposed to 2 Gy, 4 Gy, 8 Gy, and 12 Gy disposable TBI, respectively, and the median lethal dose was tested. Secondly, furazolidone was a synthetic organic compound that belongs to nitrofurans that were initially derived from furfural. It exhibited antibacterial and antihelmintic properties in humans and animals. The mice were randomly divided into 4 groups: TBI+saline, TBI+60 mg/kg FZD, TBI+100 mg/kg FZD, and TBI+140 mg/kg FZD. The FZD was given by oral gavage 30 minutes before a sublethal TBI (4 Gy) and once daily for 4 days. Mice were finally euthanized on the 15th day after TBI.

### 2.6. Analysis on Blood Cells and Micronuclei in Bone Marrow

At 15 days postirradiation, peripheral blood from the orbital sinus was collected and the number of white blood cells was analyzed. Mice were sacrificed by cervical dislocation, and the spleen and thymus were removed and weighed. The organ index was calculated according to the following formula: organ index = organ weight (g)/body weight (g). The chest cavity of mice was opened to remove the sternum, and then, the bone marrow of the sternum was squeezed with hemostatic forceps. Put a drop of bovine serum on a clean slide, mixed well and pushed the slide to dry. The bone marrow was fixed with methanol and stained with Giemsa. The number of micronuclei (MN) in 1000 polychromatic erythrocytes was counted by microscopy, and the rate of MN (‰) in bone marrow polychromatic erythrocytes was calculated.

### 2.7. Histopathology Analysis and Immunohistochemistry Staining

The intestine was removed and fixed in the formalin. Tissue was embedded in paraffin and cut into cross sections per segment in each mouse. The slides were stained with hematoxylin/eosin (H&E) for histopathology analysis by light microscopy. Gastrointestinal (GI) damage was diagnosed when the small intestines displayed denuded mucosa with no villus and no apparent crypts, respectively. Intestinal cell apoptosis was assessed in intestinal sections with TUNEL stain.

### 2.8. Western Blotting

Intestinal protein extracts were separated by 12% SDS-PAGE and transferred to nitrocellulose membranes using the Protean Mini Cell (Bio-Rad). After completion of the transfer, membranes were blocked with 5% nonfat milk in TBS/0.1% Tween 20 for 120 min. Incubation with the primary antibody (as indicated) was conducted overnight at 4°C. Incubation with a peroxidase-conjugated anti-mouse or anti-rabbit secondary antibody (1 : 2000) was performed for 120 min at room temperature. Finally, chemiluminescent analysis was performed.

### 2.9. Cell Proliferation by the CCK-8 Assay

Cell Counting Kit-8 (Dojindo Laboratories, Japan) was used according to the manufacturer's protocol. Cells were seeded in 96-well plates (3.5 × 10^3^ cells/well) and pretreated with FZD (3, 6, 12, 24, 48, 96, 192, and 384 ng/ml) followed by 8 Gy X radiation. The OD values were recorded after 2 h of CCK-8 incubation at 450 nm using a plate reader. The proliferation rate of cells was calculated by the following formula: cell viability = (OD experimental group/OD control group) × 100%.

### 2.10. Flow Cytometric Analysis of Apoptosis

Quantification of apoptotic cells was performed according to the Annexin-V/PI Apoptosis Detection Kit manufacturer's instructions (BD 556547). Analyses were performed by using a flow cytometer (ACEA NovoCyte 2040R, USA). Annexin-V-positive and PI-negative cells were regarded as apoptotic cells.

### 2.11. Analysis of Mitochondrial ROS (mtROS)

Mitochondrial ROS (mtROS) level in IEC-6 cells was detected by the MitoSOX Red (Invitrogen M36008) assay according to the manufacturer's instructions. The cells were seeded in 6-well plates with a density of 2.5 × 10^4^/ml with 3 parallel wells in each group. After various treatments, cells were stained with a MitoSOX Red probe at a final concentration of 10 *μ*M at 37°C in the dark for 15 min. Then, cells were washed with PBS three times, and the activity of mtROS was analyzed using a flow cytometer (ACEA NovoCyte 2040R, USA).

### 2.12. Cell Cycle Analysis

The cell cycle was performed by propidium iodide (PI) staining and analyzed using flow cytometry. The cells were pretreated with DMSO (SolarBio) and FZD (12 ng/ml; Sigma) for 2 h followed by radiation. After 72 h, cells were collected by trypsinization, washed with PBS, and fixed in 75% ethanol at 4°C for at least 24 hours. Cells were washed twice in PBS, and nuclear DNA was stained with 250 *μ*l propidium iodide (50 *μ*g/ml; SolarBio) in the presence of RNase A (1 *μ*l, 10 mg/ml; SolarBio) in PBS for at least 30 minutes and then subjected to flow cytometric analysis (ACEA NovoCyte 2040R, USA).

### 2.13. Statistical Analysis

Statistical analysis was performed using GraphPad Prism 5 software with the unpaired *t*-test (nonparametric) and analysis of variance (ANOVA) for data mean comparisons. Fisher's test was used for evaluating the survival of mice. Differences were considered significant at *p* < 0.05. Results are presented as means ± standard deviations.

## 3. Results

### 3.1. Furazolidone Increased the Survival of Mice Exposed to a Lethal Dose of TBI

**Figure 1:**
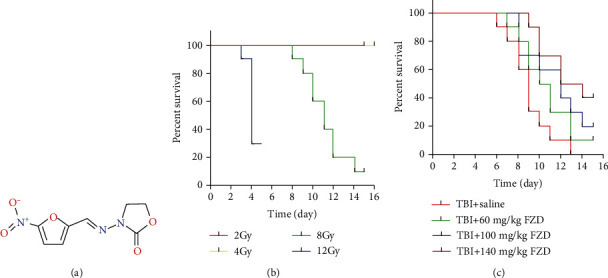
Furazolidone increased the survival time of mice exposed to a lethal dose of TBI. (a) Chemical structure of FZD. (b) Mice received different doses of total body radiation and were monitored for 15 days. (c) Effect of different concentrations of FZD on the survival time of mice exposed to a single dose of 8 Gy. Survival analysis was performed using the Kaplan-Meier curves and log-rank test.

The median lethal dose of TBI in mice was estimated by overall survival after different doses of irradiation. 40 mice were randomly averagely divided into 4 groups and, respectively, exposed to 2 Gy, 4 Gy, 8 Gy, and 12 Gy disposable TBI. The median lethal dose of TBI was 6 Gy (Figures 1(a) and 1(b)). To identify the dose of FZD on the survival time of irradiated mice, we conducted the postirradiation 15-day survival trial. Mice were also divided into 4 groups and treated with TBI+saline, TBI+60 mg/kg FZD, TBI+100 mg/kg FZD, and TBI+140 mg/kg FZD by intragastric administration. Mice were exposed to a lethal dose (8 Gy, for the survival time study only) of TBI. Irradiated mice treated with FZD exhibited a better physical situation than those without the treatment. FZD administration significantly improved the survival time of irradiated mice; moreover, higher FZD treatment showed better effect (Figure 1(c)).

### 3.2. Furazolidone Improved the Immune and Hematopoiesis Index in Mice Exposed to a Lethal Dose of TBI

**Figure 2:**
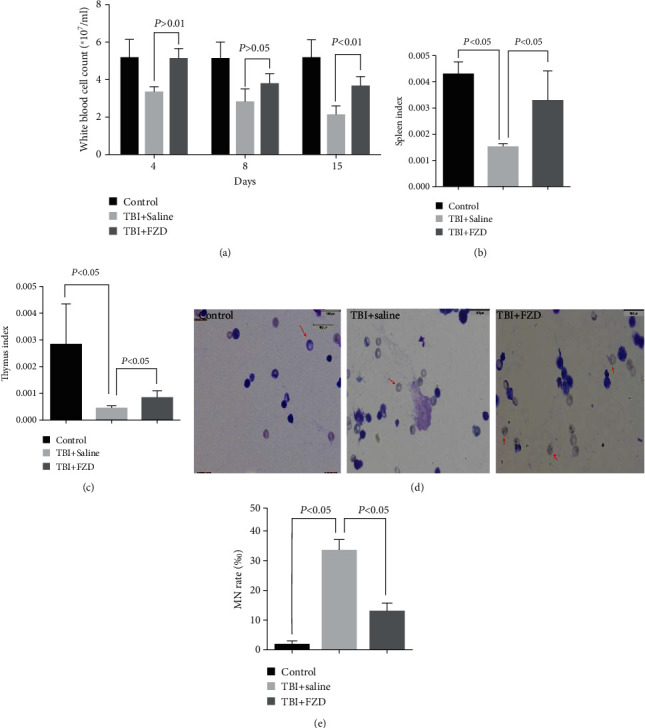
FZD protected against IR-induced hematopoiesis and immunity injury. (a) White blood cell count of mice exposed to a lethal dose of TBI was calculated at different time points (D4, D8, and D15). (b) The splenic index. (c) The thymus index. (d, e) The number of MN in the bone marrow was detected with a microscope, and the MN rate (‰) in bone marrow polychromatic erythrocytes was calculated. Error bars are means ± SD (*n* = 3 independent repeats), and *p* values were calculated using two-tailed unpaired Student's *t*-test.

To investigate whether FZD protects mice from TBI-induced hematopoietic injury, the peripheral blood cell counts were measured at 15 d after 4 Gy TBI. Compared with saline-treated mice, the numbers of WBCs exhibited a clear increase in FZD-administrated mice (Figure 2(a)). To determine whether FZD treatment protected mice from IR-induced organ index changes, the spleen index and thymus index were measured, and FZD attenuated the decrease in the spleen index and thymus index following radiation treatment (Figures 2(b) and 2(c)). IR induces unstable chromosomal aberrations such as acentric and dicentric chromosomes and chromosome breaks, those fragments are unable to interact with the spindle poles during mitosis, and as a result, one or several micronuclei (MN) are formed [11]. Since bone marrow is one of the most radiosensitive tissues [12], the number of MN in the bone marrow is a good indicator of radiation exposure and DNA damage [13]. The number of MN was upregulated compared with the control group after TBI, and FZD rescued the decrease in MN (Figures 2(d) and 2(e)).

### 3.3. FZD Restored Intestinal Integrity in Mice Exposed to a Lethal Dose of TBI

**Figure 3:**
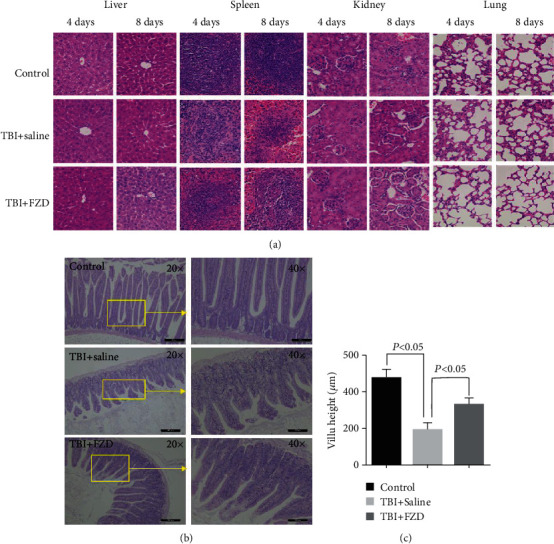
FZD restored intestinal integrity in mice exposed to a lethal dose of TBI. (a) Histology analysis of different tissues (liver, spleen, kidney, and lung) in mice at different time points (D4 and D8) post-8 Gy radiation. Sections were stained with hematoxylin and eosin, and histological examination was applied. (b) HE staining of intestinal tissue in mice; upper 20x and lower 40x. (c) Intestinal villus height. Error bars are means ± SD (*n* = 3 independent repeats), and *p* values were calculated using two-tailed unpaired Student's *t*-test.

In order to detect the target tissue which FZD protected against radiation damage, the liver, spleen, kidney, and lung tissues were taken for hematoxylin-eosin staining. Compared with the control group, liver, spleen, kidney, and lung tissues failed to change after TBI (Figure 3(a)). Since TBI can lead to the onset of the gastrointestinal syndrome, which is characterized by loss of the lining of the intestine, necrosis of tissue in the gastrointestinal tract and villi shortening necrosis of gastrointestinal tissue and shortening of villi in small intestine. [3, 12]. Villi were observed in the intestines of mice pretreated with saline or FZD. Irradiation resulted in villus shortening and crypt dilating accompanied by epithelial atrophy or slough and even marked edema and inflammatory cell infiltration. FZD significantly recovered the damage (Figures 3(b) and 3(c)). To further verify whether FZD could rescue cell apoptosis induced by ionizing radiation, the flow cytometry assay was applied.

### 3.4. Furazolidone Treatment Inhibited IR-Induced Apoptosis and Autophagy in the Small Intestine

**Figure 4:**
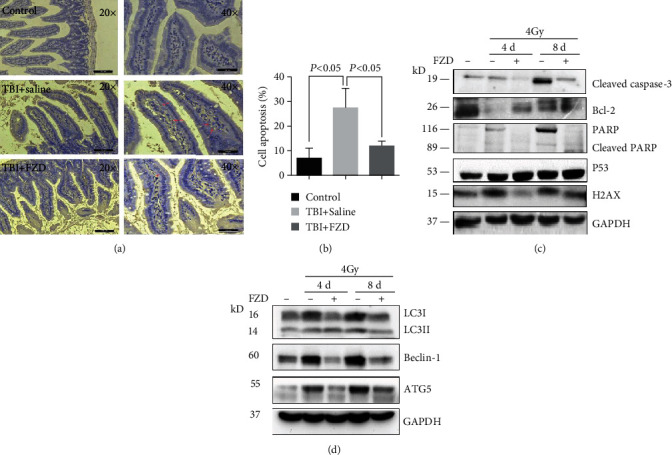
FZD treatment reduced apoptosis and autophagy in villi. (a) TUNEL staining of intestinal tissue sections. (b) The calculated statistical data from TUNEL staining. (c) Western blot analysis of apoptosis in intestinal tissue. Cleaved caspase-3, Bcl-2, PARP, p53, and H2AX were included. (d) Western blot analysis of autophagy in intestinal tissue of mice. MAPLC3, beclin-1, and ATG5 were included. Error bars are means ± SD (*n* = 3 independent repeats), and *p* values were calculated using two-tailed unpaired Student's *t*-test.

Since the villi of the intestine were significantly shortened following radiation treatment, FZD preferred to enrich in small intestine. We further detected whether FZD has a radiation protection effect on the intestine tissue. Previous studies have shown that exposure to IR provokes DNA damage and triggers various forms of cell deaths [4, 5]. Then, the TUNEL staining assay was applied for apoptosis. As depicted in (Figures 4(a) and 4(b)), FZD treatment resulted in a marked recovery in the percentage of apoptotic cells, suggesting the roles of FZD in the process of antiapoptosis induced by TBI. To explore the apoptosis alteration more comprehensively, we detected the intestine proteins and found significant decrease in cleaved caspase-3, cleaved PARP, and H2AX expression and increase in Bcl-2 and p53 expression after FZD treatment (Figure 4(c)). These results indicated that TBI could induce apoptosis in intestine tissue, and FZD could reverse this phenomenon. The activation of autophagy in normal tissue following stress, such as radiation, may rely in part on the interplay between apoptosis and autophagy [14]. Most studies describe beclin-1 and Bcl-2 as key proteins in the interplay between these two cellular processes [15, 16]. So, we determined the expression of beclin-1 protein in the intestine of mice. Western blot demonstrated that FZD could reduce IR-induced increase in beclin-1. Then, we tested other autophagy-related proteins by western blot (Figure 4(d)), compared to the IR group. Both of ATG5 expression and the ratio of LC3II/I were decreased in the FZD+IR group.

### 3.5. Furazolidone Treatment Decreased Radiosensitivity of IEC-6 Cells

**Figure 5:**
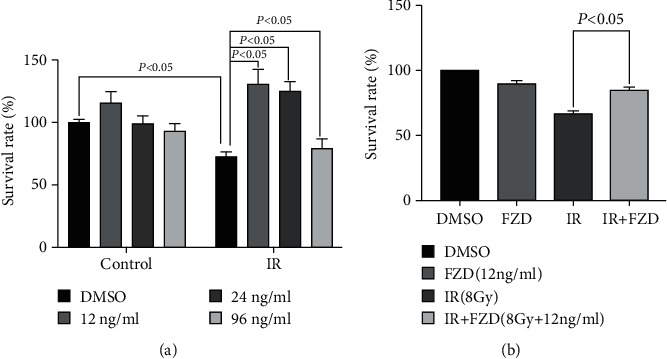
Protective effect of FZD in IR-induced cell death in IEC-6 cells. (a) The CCK-8 assay. (b) The trypan blue assay. For each concentration point, three repeats were performed. Error bars are means ± SD (*n* = 3 independent repeats), and *p* values were calculated using two-tailed unpaired Student's *t*-test.

To further investigate the protective effect of FZD, we then designed in vitro experiments and an intestinal epithelial cell line, IEC-6, was used to determine the underlying mechanisms. After 12 ng/ml FZD treatment, cell survival significantly increased compared with the DMSO group by the CCK-8 assay (Figure 5(a)). Also, a significant increase in cell survival was observed in the FZD+IR group as compared with the IR group, and no significant changes occurred in the FZD group with the DMSO group by the trypan blue assay (Figure 5(b)). These data suggest that FZD could reverse IR-induced cell death in IEC-6 cells.

### 3.6. Furazolidone Increased IR-Induced Cell Cycle Arrest in IEC-6

**Figure 6:**
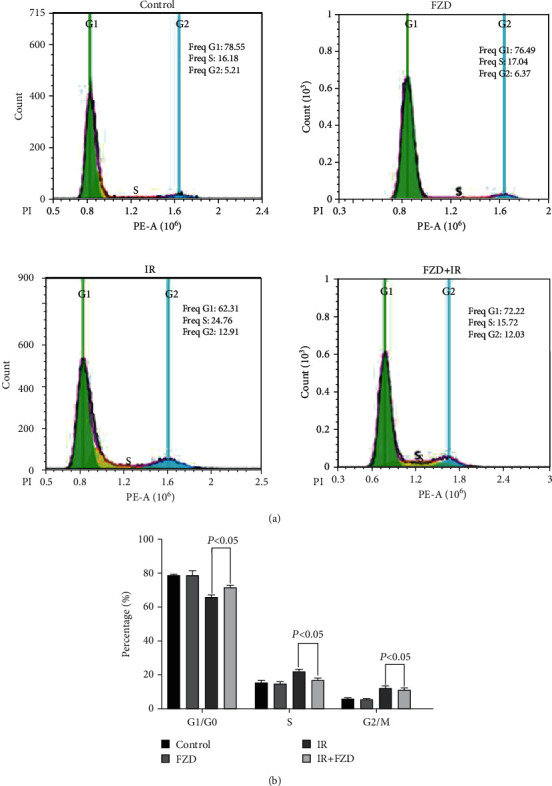
Furazolidone could attenuate the cell cycle arrest of IEC6 caused by radiation. (a) Cell cycle was determined by FACS analysis following propidium iodide staining. The cells were pretreated with FZD for 2 h following 8 Gy radiation for 72 h. (b) The graph shows the percentage of cells in the cell cycle. Error bars are means ± SD (*n* = 3 independent repeats), and *p* values were calculated using two-tailed unpaired Student's *t*-test. The graph shows the percentage of cells on the S phase.

Ionizing radiation can cause DNA damage in cells and then cause a series of complex reactions, including cell cycle arrest, DNA repair, and apoptosis. To further investigate whether FZD had an effect on radiation-induced cell cycle arrest in IEC-6 cells, IEC6 cells were divided into four groups: DMSO, FZD (12 ng/ml), IR+DMSO, and IR+FZD (12 ng/ml). The cells in the G0/G1 phase markedly decreased, and the cells in the S phase and G2/M phase accumulated in the IR group. In the IR+FZD group, the cells in the G1/G0 phase were significantly increased and the cells in the S phase and G2/M phase were decreased, indicating that FZD induced G0/G1 cell cycle arrest and delayed the cell cycle process, which is beneficial to damage repair. Also, FZD inhibited the S phase delay, which is beneficial to decreasing the occurrence of polyploid giant cells caused by S phase uncoupling and reducing the risk of cell transformation (Figures 6(a) and 6(b)).

### 3.7. FZD Inhibited IR-Induced Apoptosis and Autophagy of IEC-6 Cells

**Figure 7:**
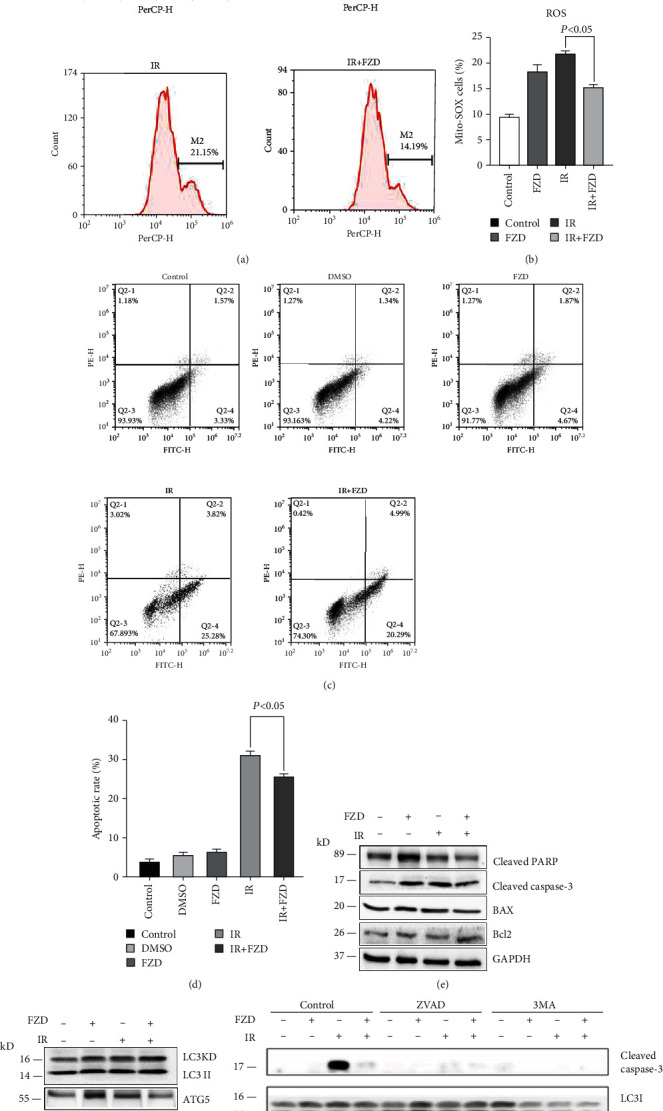
FZD reduced IR-induced apoptosis and autophagy in IEC-6. (a) A dual-staining with annexin V and PI followed by the flow cytometry assay was used to examine IEC-6 cell apoptosis. Error bars are means ± SD (*n* = 3 independent repeats), and *p* values were calculated using two-tailed unpaired Student's *t*-test. (b) Flow cytometric analysis of IEC-6 cells for mtROS with MitoSOX. Error bars are means ± SD (*n* = 3 independent repeats), and *p* values were calculated using two-tailed unpaired Student's *t*-test. (c) The expression of cleaved PARP, cleaved caspase-3, BAX, and Bcl-2 by western blot in IEC-6 following different treatments. (d) The expression of MAPLC3, beclin-1, and ATG5 in IEC-6 following different treatments. (e) The expression of caspase-3 and LC3 following different treatments.

Because radiation can directly or indirectly increase ROS levels in cells, ROS-induced apoptosis is one of the main mechanisms of radiation. FZD had a protective effect against the radiation-induced cell death, and the mechanism may be related to reducing ROS release and inhibiting the ROS/caspase-3 signaling pathway. To determine whether FZD had the protective effect through ROS release and apoptosis, the ROS level and apoptosis were detected. As shown in Figures 7(a)–7(d), the ROS content and apoptosis in IR combined with FZD cells were significantly reduced, suggesting an important role of FZD in the process of anti-ROS and anti-apoptosis induced by IR. To further clarify the underlying mechanism on the protective effects of FZD, the apoptosis- and autophagy-related proteins were detected after radiation. Western blot data showed that FZD significantly decreased the expression of cleaved caspase-3 and BAX and increased the expression of Bcl-2 (Figure 7(e)); FZD also decreased the radiation-induced expression of LC3, ATG5 and beclin-1 (Figure 7(f)), suggesting the regulatory and protective roles of FZD via apoptosis and autophagy processes. To further elucidate the crosstalk between apoptosis and autophagy in this situation, as shown in (Figure 7(g)), ZVAD and 3MA significantly decreased the expression of cleaved caspase-3 suggesting that autophagy might promote apoptosis. Inhibition of the apoptosis by ZVAD also can promote autophagy, but the underlying mechanism should be further studied.

## 4. Discussion

It is well known that a higher dose of TBI could cause ARS in which three syndromes were included: hematopoietic bone marrow syndrome, gastrointestinal syndrome, and brain syndrome [17, 18]. So far, effective radioprotectors are still limited and few have been applied. FZD is routinely used as a medicinal herb to treat various diseases, and the therapeutic potential of FZD as a radioprotector is poorly elucidated. In this study, we found that FZD improved the survival of mice suffering from lethal dose of TBI and reversed IR-induced injury represented by the spleen index, thymus index, and MN of bone marrow, suggesting that FZD might play a protective role in IR-induced organ injury. The amount of MN could reflect the degree of DNA damage, and FZD treatment reduced the MN in BM, suggesting that FZD influences the process of DNA repair after IR.

Since the primary pathological changes in bone marrow syndrome are the suppression of bone marrow hematopoietic functions, including the decrease in peripheral blood WBCs [19], we analyzed the WBC count and found that FZD treatment could increase the number of WBCs, suggesting that FZD improves the bone marrow hematopoietic functions.

Since the intestine is one of the most radiosensitive organs, intestinal injury is a very common complication of radiotherapy [12]. AGS starts from mucosal epithelium injury and finally leads to the loss of gastrointestinal function. After irradiation, progressive necrosis and shedding of the intestinal villus epithelium, villus atrophy, and cell gap expansion could be detected [20]. We found that FZD could significantly restore intestinal integrity, such as villi and crypts after TBI.

Previous studies have shown that exposure to IR provokes DNA damage and triggers various forms of cell deaths [5]. Our results also showed that TBI increased apoptosis and autophagy in the small intestine, which could be rescued by FZD. Proapoptotic signaling pathways are known to involve the activation and stabilization of Bcl-2, Bax, caspase-3, P21 and more signaling molecules [21]. The Bcl-2 family includes proapoptotic and antiapoptotic molecules: Bcl-2 is an antiapoptotic protein and Bax is a proapoptotic protein. They are considered to be major regulatory factors, and the ratio of Bcl-2/Bax plays an important role in apoptosis. Our results suggested that FZD could inhibit IR-induced apoptosis of the intestinal epithelium. Compared with the IR-alone group, the adding of FZD further increased the radiation-induced P53 expression in intestinal epithelia and consequently decreased the radiation-induced damage to the intestine. The above-mentioned data is consistent with the data in Lee et al.'s paper. Lee et al. found that P53 could contribute to death or survival in a cell type-dependent manner, which underscores the complexity by which p53 regulates the cellular and tissue response to radiation [22]. The expression of autophagy-related genes beclin-1, MAPLC3 I/II, and ATG5 increased following TBI treatment, which could be reversed by FZD. Those results suggested that FZD could inhibit IR-induced autophagy of the intestinal epithelium.

In vitro experiments were then used to verify the underlying mechanisms. The data showed that FZD inhibited IEC-6 death caused by irradiation. FZD maintained most of cells in the G0/G1 phase and delayed the cell cycle process, which is beneficial to damage repair. Also, FZD inhibited the S phase delay, which is beneficial to decreasing the occurrence of polyploid giant cells caused by S phase uncoupling and reducing the risk of cell transformation. Our results showed that FZD inhibited radiation-induced ROS generation, apoptosis, and autophagy. In addition, FZD treatment reduced the protein expression of MAP LC3II/LC3I, beclin-1, and ATG5 following radiation treatment, suggesting that FZD could also inhibit radiation-induced autophagy. However, the molecular mechanism of how FZD protects cells from autophagy and apoptosis remains to be further studied.

In summary, FZD treatment can improve the survival of mice treated with lethal dose radiation by alleviating TBI-induced injury and inhibiting autophagy and apoptosis. FZD is a clinically used drug with few side effects and might be used as an efficacious medical radiation countermeasure. However, the detailed radiation protection activity, clinical application, molecular mechanism, and drug target of FZD need to be further studied.

## Data Availability

All data generated or analyzed during this study are included in this article.

## Abbreviations

TBI: Total body irradiation
FZD: Furazolidone
IR: Ionizing radiation
ARS: Acute radiation syndrome
DBS: Double-strand breaks
ROS: Reactive oxygen species
AGS: Acute gastrointestinal syndrome
MN: Micronuclei
H&E: Hematoxylin/eosin
GI: Gastrointestinal
PI: Propidium iodide
WBCs: White blood cells
BM: Bone marrow
ARS: Acute radiation syndrome.
